# Serotonin syndrome by drug interactions with linezolid: clues from pharmacovigilance-pharmacokinetic/pharmacodynamic analysis

**DOI:** 10.1007/s00228-020-02990-1

**Published:** 2020-09-08

**Authors:** Milo Gatti, Emanuel Raschi, Fabrizio De Ponti

**Affiliations:** Pharmacology Unit, Department of Medical and Surgical Sciences, Alma Mater Studiorum, University of Bologna, Via Irnerio, 48, 40126 Bologna, Italy

**Keywords:** Linezolid, Serotonin syndrome, Drug-drug interactions, Pharmacovigilance-pharmacokinetic/pharmacodynamic approach, Infectious disease “dilemma”

## Abstract

**Purpose:**

To characterize the post-marketing reporting of serotonin syndrome (SS) due to drug-drug interactions (DDIs) with linezolid and investigate the relationship with pharmacokinetic/pharmacodynamic (PK/PD) properties of serotonergic agents.

**Methods:**

We queried the worldwide FDA Adverse Event Reporting System to extract SS records due to DDIs where linezolid was reported as suspect. For each serotonergic agent concomitantly reported, proportion of SS reports and mean number of DDIs were calculated and three different “SS reporting zones” were created. Relevant PK (peak concentration, area under plasma concentration curve, volume of distribution (V_D_), and lipophilicity) and PD (values of binding affinity (Ki) and IC_50_ for serotonin reuptake transporter (SERT) and 5-HT_2A_) parameters were extracted for each serotonergic agent, and relevant PK/PD indexes were calculated to assess correlation with mean number of DDIs (PV index).

**Results:**

Six hundred sixty-nine reports of SS mentioning linezolid were found, being linezolid-citalopram (*N* = 69; 10.3%) the most frequently DDI reported. Citalopram and methadone showed respectively the highest proportion of SS reports (0.28%) and the lowest mean number of DDIs (1.41). Citalopram, escitalopram, and methadone emerged as red (i.e., alert)-zone medications: they exhibited high lipophilicity and large V_D_ (proxies of excellent central nervous system penetration) coupled with high potency. Among PK/PD indexes, a significant correlation with PV index was found for V_D_/Ki SERT ratio (*p* = 0.05).

**Discussion:**

Our integrated approach suggests that linezolid is more likely to cause SS when co-administered with citalopram, escitalopram, and methadone, as inferred from their pharmacological properties. Proper management of SS should be tailored on a case-by-case basis.

**Electronic supplementary material:**

The online version of this article (10.1007/s00228-020-02990-1) contains supplementary material, which is available to authorized users.

## Introduction

Linezolid is an oxazolidinone antibiotic with activity against multidrug-resistant Gram-positive organisms [1], showing lipophilic features, excellent tissue penetration including the central nervous system (CNS), and weak reversible non-selective monoamine oxidase (MAO) inhibitory effects at therapeutic serum concentrations (according to an inhibitory binding affinity constant (Ki) of 56 μM and 0.71 μM, respectively for MAO-A and MAO-B) [2, 3]. MAO is involved in the metabolism of the monoamine neurotransmitters, and its inhibition may potentially lead to excess of serotonin (5-hydroxytriptamine (5-HT)) in the CNS and occurrence of serotonin syndrome (SS), a potential life-threatening condition [4, 5]. Inhibition of the serotonin reuptake transporter (SERT), causing accumulation of serotonin in the synaptic cleft and overstimulation of 5-HT_2A_ receptors, is supposed to be the main mechanism involved in SS [5].

**Fig. 1:**
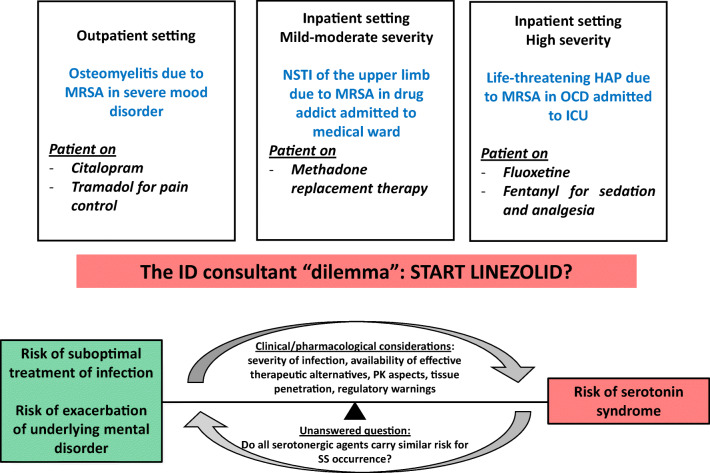
Examples of clinical scenarios posing a “dilemma” to the infectious disease consultant regarding the use of linezolid in patients on serotonergic agents (MRSA, methicillin-resistant *Staphylococcus aureus*; NSTI, necrotizing soft tissue infection; HAP, hospital-acquired pneumonia; OCD, obsessive-compulsive disorder; ICU, intensive care unit; ID, infectious disease; PK, pharmacokinetic; SS, serotonin syndrome)

Several drugs may cause serotonin excess through different pathways [6], thus posing concerns when concomitant treatments with linezolid are needed. Although reports describing occurrence of SS due to drug-drug interactions (DDIs) with linezolid were reported [3, 7, 8], and the Food and Drug Administration (FDA) warned against co-administration of linezolid with serotonergic drugs recommending a 2-week washout period in patients already receiving these medications [9], treatment of life-threatening infections may not be delayed, especially if second-line alternatives are not effective or available. However, it remains unclear whether all serotonergic agents carry similar risk of SS, and the infectious disease consultant faces a “dilemma,” with relevant implications on rational drug use in specific clinical scenarios, as exemplified in Fig. 1.

In order to address this aspect and provide clues to help in decision-making, we investigated the relationship between real-world data, namely, spontaneous reports submitted to the US FDA adverse event reporting system (FAERS) database, and pharmacokinetic/pharmacodynamic (PK/PD) properties of serotonergic agents.

## Materials and methods

We carried out a “two-step” approach to investigate whether the reporting pattern of SS induced by linezolid is potentially related to the burden of co-medications resulting in clinically significant DDIs, and relevant PK/PD properties of serotonergic agents, an emerging approach recently proposed to assess the pharmacological basis of adverse events (AEs) observed in a worldwide pharmacovigilance (PV) database [10–12].

First, the FAERS public dashboard database, the US repository of AEs and medication errors comprising more than 18 million reports gathered worldwide, was queried to retrieve SS reports recorded until December 2019, using “serotonin syndrome” as preferred term, to extract only those records where linezolid was reported as suspect. In order to select potential DDIs, co-administered serotonergic agents reported as suspect were identified according to the lists proposed by Bower et al. [6] and Woytowish et al. [13]. Post-marketing studies using the FAERS are effective for continuous monitoring of old medications [14], including the analysis of potential DDIs [15], with noteworthy performance (i.e., the capacity to discriminate true from positive drug-event associations) [16], especially for side effects with low/rare background incidence and a likely drug-attributable component such as SS.

For each serotonergic agent concomitantly reported with linezolid in ≥ 5 cases of SS, two indexes were created: (a) proportion of SS reports, as compared with the overall number of reports, and (b) mean number of DDIs, based on concomitant serotonergic agents recorded in SS reports, being 1 the value corresponding to the single interaction between linezolid and the drug of interest. A scatterplot showing the relationship between the two indexes for each serotonergic agent was created. Threshold values of ≥ 0.1% and ≤ 1.5 were respectively selected for proportion of SS reports (according to 0.09% incidence of SS in a retrospective study including patients exposed to serotonergic medications [17]) and mean number of DDIs (based on the estimation that in almost half of records, no other concomitant serotonergic agent was reported, except for drug of interest), thus identifying three different “SS reporting zones”:

- “red (i.e., alert)-zone,” including medications with high proportion of SS reports and low mean number of DDIs
- “yellow-zone,” including medications with high proportion of SS reports coupled with high mean number of DDIs or low proportion of SS reports coupled with low mean number of DDIs
- “green-zone,” including medications with low proportion of SS reports and high mean number of DDIs

In the second step, physiochemical/PK (peak concentration (C_max_), area under plasma concentration curve (AUC), volume of distribution (V_D_), and lipophilicity (LogP)) and PD (values of Ki and IC_50_ (concentration corresponding to 50% inhibition of activity in vitro) for SERT and 5-HT_2A_) data of each selected serotonergic agents were extracted from MEDLINE/PubMed and DrugBank databases [18] (searches performed on April 22, 2020). For PK parameters, the highest mean value reported for each medication when administered at therapeutic dosage was selected. The following PK/PD indexes were created: C_max_/K_i_ SERT, C_max_/IC_50_ SERT, C_max_/K_i_ 5-HT_2A_, AUC/K_i_ SERT, AUC/IC_50_ SERT, AUC/K_i_ 5-HT_2A_, V_D_/K_i_ SERT, V_D_ /IC_50_ SERT, V_D_/K_i_ 5-HT_2A_, LogP/K_i_ SERT, LogP/IC_50_ SERT, and LogP/K_i_ 5-HT_2A_.

Correlation between PK/PD indexes and mean number of DDIs (PV index) was assessed by calculating Spearman’s rank correlation coefficient, considering significant a *p* value < 0.05.

## Results

Overall, 11,429 reports of SS were found, 669 (5.9%) of which mentioning linezolid as suspect agent. Mean age was 55 ± 26.3 years, with no gender preponderance; 99.1% of reports were serious (i.e., resulting in death, hospitalization, or life-threatening event), and death was reported in 41 cases (18 with citalopram co-administration).

Single DDI was reported in 366 cases (54.7%), being linezolid-citalopram (*N* = 69; 10.3%) and linezolid-fentanyl (46; 6.9%) the most frequent, while multiple DDIs were reported in 179 cases (26.8%), being linezolid-citalopram-tramadol (23; 3.4%) the predominant (Supplementary Table 1). In 124 reports (18.5%), no concomitant serotonergic medications were recorded. Citalopram (*N* = 112), fentanyl (*N* = 100), sertraline (*N* = 74), escitalopram (*N* = 65), and tramadol (*N* = 51) were the most frequently serotonergic agents mentioned as suspect with linezolid in cases of SS.

**Fig. 2:**
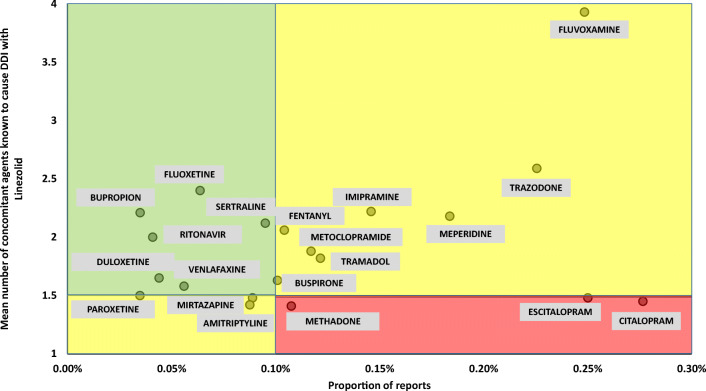
Scatterplot showing the relationship between the proportion of SS reports (on *x*-axis) and the mean number of DDIs (on *y*-axis) for each serotonergic agent. Threshold values of ≥ 0.1% and ≤ 1.5 were respectively selected for proportion of SS reports and mean number of DDIs, identifying three different SS risk zones (red-zone, high-risk medications; yellow-zone, intermediate-risk medications; green-zone, low-risk medications)

Citalopram and methadone showed respectively the highest proportion of SS reports (0.277%) and the lowest mean number of DDIs (1.41; Supplementary Table 2). Citalopram, escitalopram, and methadone emerged as red-zone medications (Fig. 2). Additionally, amitriptyline (1.42), mirtazapine (1.48), and paroxetine (1.5) also exhibited a mean number of DDIs ≤ 1.5, although classified as “yellow-zone” agents due to their low proportion of SS reports.

A summary of PK/PD properties of serotonergic medications is shown in Supplementary Table 3. Among antidepressants, venlafaxine and sertraline showed respectively the highest V_D_ and the lowest IC_50_ for SERT, while methadone exhibited both the highest V_D_ and LogP, as well as the lowest IC_50_ for SERT among opioids.

**Fig. 3:**
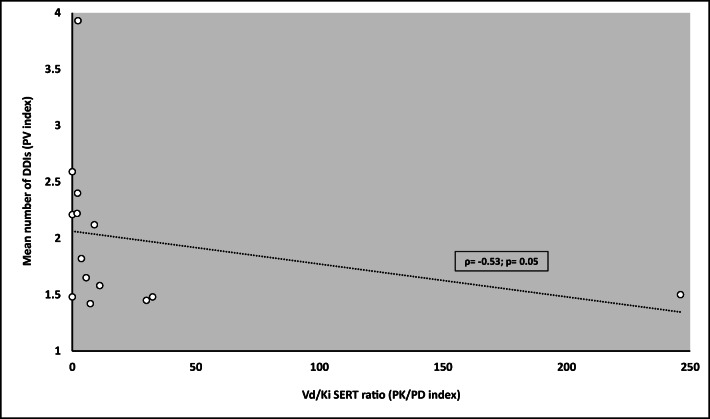
Scatterplot showing the relationship between VD/Ki SERT ratio (PK/PD index; *x*-axis) and mean number of DDIs (PV index; *y*-axis). A significant correlation was found (*ρ* = − 0.53; *p* = 0.05)

A significant correlation between PK/PD and PV indexes was found for the V_D_/Ki SERT ratio (*ρ* = − 0.53; *p* = 0.05; Fig. 3), while a trend was observed for the V_D_/IC_50_ SERT (*ρ* = − 0.405; *p* = 0.11), LogP/K_i_ SERT (*ρ* = − 0.504; *p* = 0.07), and LogP/IC_50_ SERT ratios (*ρ* = − 0.399; *p* = 0.09; Table 1). No significant correlation emerged between PK/PD indexes investigating plasma exposure of serotonergic agents (namely, C_max_ and AUC) or binding affinity for 5-HT_2A_ and PV index (Table 1).

**Table 1:** Summary of correlation between PK/PD indexes (independent variable) and PV index (mean number of DDIs; dependent variable)

PK/PD – PV correlation	*ρ* value (95% CI)	*p* value	Interpretation
C_max_/K_i_ SERT – mean number of DDIs (*N* = 14)	− 0.334 (− 0.735–0.238)	0.24	No significant correlation
C_max_/IC_50_ SERT – mean number of DDIs (*N* = 19)	− 0.20 (− 0.60–0.28)	0.41	No significant correlation
C_max_/K_i_ 5-HT_2A_ – mean number of DDIs (*N* = 17)	0.002 (− 0.479–0.483)	0.99	No significant correlation
AUC/K_i_ SERT – mean number of DDIs (*N* = 14)	− 0.374 (− 0.755–0.195)	0.19	No significant correlation
AUC/IC_50_ SERT – mean number of DDIs (*N* = 18)	− 0.376 (− 0.717–0.11)	0.12	Trend to significant correlation (agents with high exposure on time and high potency on SERT are more likely to cause SS when used individually with linezolid)
AUC/K_i_ 5-HT_2A_ – mean number of DDIs (*N* = 17)	− 0.134 (− 0.577–0.371)	0.61	No significant correlation
V_D_/K_i_ SERT – mean number of DDIs (*N* = 14)	− 0.53 (− 0.828–0.00)	0.05	Significant correlation (agents with high V_D_ and high potency on SERT are more likely to cause SS when used individually with linezolid)
V_D_ /IC_50_ SERT – mean number of DDIs (*N* = 17)	− 0.405 (− 0.741–0.094)	0.11	Trend to significant correlation (agents with high V_D_ and high potency on SERT are more likely to cause SS when used individually with linezolid)
V_D_/K_i_ 5-HT_2A_ – mean number of DDIs (*N* = 17)	− 0.163 (− 0.597–0.345)	0.53	No significant correlation
LogP/K_i_ SERT – mean number of DDIs (*N* = 14)	− 0.504 (− 0.816–0.037)	0.07	Trend to significant correlation (agents with high lipophilicity and high potency on SERT are more likely to cause SS when used individually with linezolid)
LogP/IC_50_ SERT – mean number of DDIs (*N* = 19)	− 0.399 (− 0.722–0.067)	0.09	Trend to significant correlation (agents with high lipophilicity and high potency on SERT are more likely to cause SS when used individually with linezolid)
LogP/K_i_ 5-HT_2A_ – mean number of DDIs (*N* = 17)	− 0.123 (− 0.57–0.38)	0.64	No significant correlation

Spearman’s rank correlation coefficient was calculated, and a *p* value < 0.05 was considered significant

*N* number of serotonergic medications for which specific PK/PD parameters are retrieved, *PK* pharmacokinetic, *PD* pharmacodynamic, *PV* pharmacovigilance, *C*_*max*_ peak concentration, *AUC* area under plasma concentration curve, *V*_*D*_ volume of distribution, *Ki* binding affinity, *IC*_*50*_ concentration corresponding to 50% inhibition of activity in vitro, *SERT* serotonin reuptake transporter, *DDIs* drug-drug interactions, *SS* serotonin syndrome

## Discussion

Our real-world study, by investigating the worldwide reporting of SS with linezolid and concomitant serotonergic drugs and analyzing their potential contributing role in the light of relevant PK/PD properties, provides some clues towards safer prescribing. Although previously proposed [10–12], to the best of our knowledge, this is the first study in which this PV-PK/PD approach combined multiple sources of data and also accounted for the contributing role of DDIs.

Citalopram, escitalopram, and methadone emerged as “red-light drugs” according to PV indexes, given the high proportion of reports coupled with their potential to precipitate SS when mainly administered with linezolid. Notably, these findings are closely correlated with PK/PD features, thus confirming the role of SERT inhibition as key molecular pathway involved in SS [5, 11] and suggesting that agents with high binding affinity (low Ki or IC_50_) for this transporter and large V_D_ or LogP (high lipophilicity to effectively cross the blood-brain barrier) are more likely to precipitate the disease [19]. Consequently, it is expected that agents showing a low mean number of DDIs should exhibit a large V_D_/Ki SERT ratio (high CNS penetration and high potency), as found in our analysis. This is the case of citalopram, escitalopram, and methadone, which, by virtue of their PK/PD properties, are more likely to cause SS when used individually with linezolid, in contrast with other serotonergic agents, which were reported in SS cases only under polypharmacy regimen [20–22]. Our results are in line with those of recently proposed systematic bioinformatics approach [23], which also indicated SERT inhibition by methadone as a key mechanistic link to SS. Conversely, our findings showed non-significant correlation between C_max_, AUC, and PV indices, as well as 5-HT_2A_ seams to play a minor role in comparison to SERT in the occurrence of SS. Consequently, our analysis of the different proposed PK/PD indexes likely suggested that penetration in the CNS of a specific serotonergic agent, coupled with its potency on SERT inhibition, could be more important than plasma exposure in precipitating SS when co-administered with linezolid.

Additionally, different implications could be suggested for the two “yellow-zones.” Particularly, the one including agents with low proportion of SS reports and low mean number of DDIs, namely, paroxetine, amitriptyline, and mirtazapine, could be considered as a zone exhibiting an intermediate reporting risk between the other “yellow-zone” (including agents with high proportion of SS reports and high mean number of DDIs) and the “red-zone”, due to the ability of these serotonergic medications in precipitating SS mainly when co-administered with linezolid in absence of other serotonergic drugs.

The regulatory recommendation of a reasonable washout period when starting linezolid after serotonergic agents poses a clinical challenge when prompt treatment of serious infections is needed. Given its potent activity against multidrug-resistant Gram-positive pathogens and excellent tissue penetration [24], linezolid represents the first-line agent for management of several deep-seated infections (i.e., pneumonia, meningitis, osteomyelitis) for which potential alternatives (namely, daptomycin, ceftaroline, ceftobiprole) may exhibit PK disadvantages [25, 26].

We put forward four main determinants to be considered in tailored risk-benefit assessment depending on clinical scenario: (1) color zone of the different serotonergic medications according to PV indexes and PK/PD properties, (2) severity of infection, (3) availability of effective therapeutic alternatives, and (4) risk of worsening of underlying mental disorder.

In the setting of an intubated and deeply sedated critical patient requiring treatment of a life-threatening infection, antidepressant therapy could be abruptly withdrawn, and sedation with extemporaneous or long-term administration of fentanyl or meperidine (yellow-zone medications) could be replaced with morphine. Conversely, discontinuing citalopram/escitalopram in a patient affected by severe mood disorder requiring prolonged treatment with linezolid for a spondylodiscitis could be challenging. In this scenario, exacerbation of mental illness and suicide risk could exceed the likelihood of SS occurrence, thus requiring close monitoring while maintaining both treatments. Similarly, linezolid should be promptly initiated under strict monitoring in treatment of severe pneumonia despite concomitant methadone replacement therapy, according to severity of infection and the risk for opiate withdrawal syndrome in case of methadone discontinuation.

We acknowledge the limitations of this study, related to both pharmacovigilance analyses, including FAERS data (e.g., quality of reports, potential existence of remaining duplicates, reporting biases, lack of exposure data, inability in establishing firm causality between drug exposure and occurrence of SS, absence of data to calculate the time to onset from drug administration in the public FAERS dashboard, and limited verification of events through clinical features [27, 28]), and pharmacokinetic-pharmacodynamic assessment. Additionally, absolute incidence of SS cannot be inferred by analyzing FAERS data. Consequently, although we calculated proportion of SS reports for each serotonergic agent, our index was not intended to assess absolute incidence of SS, but it would only provide a relative proportion as a percentage of overall reports for a specific agent. Moreover, SS may be underdiagnosed due to lack of recognition, thus likely increasing the extent of under-reporting, a well-known phenomenon. Furthermore, heterogeneity of PK/PD studies in assessing the different parameters have to be considered. Particularly, the value of V_D_ may be affected by serotonergic agents subjected to high pre-systemic extraction after oral dosage. However, a trend to significant correlation was also found for indexes correlating lipophilicity (LogP) and binding affinity to SERT (Ki or IC_50_), thus supporting the hypothesis that agents with high lipophilicity and penetration in CNS coupled with high potency on SERT are more likely to precipitate the disease when used individually with linezolid.

In summary, citalopram, escitalopram, and methadone emerged as “red-light medications,” according to their PV indices. High SERT inhibitory potency coupled with large V_D_ and high lipophilicity, proxies for excellent CNS penetration, may explain the role of “red-light drugs” in precipitating SS when individually co-administered with linezolid. Proper management of SS, including drug discontinuation and/or strict monitoring, should be tailored on a case-by-case basis.

## Electronic supplementary material

ESM 1 (DOCX 29 kb)

## Data Availability

Data supporting the findings of this study were derived from the following resource available in the public domain: https://fis.fda.gov/sense/app/d10be6bb-494e-4 cd2-82e4-0135608ddc13/sheet/7a47a261-d58b-4203-a8aa-6d3021737452/state/analysis.
