# Supplementary material for: Serotonin syndrome by drug interactions with linezolid: clues from pharmacovigilance-pharmacokinetic/pharmacodynamic analysis
Source: Eur J Clin Pharmacol. 2020 Sep 8;77(2):233–9. doi: 10.1007/s00228-020-02990-1 (PMC7803711; doi:10.1007/s00228-020-02990-1)
Supplement: Supplementary file 1 — (DOCX 29 kb) [file 228_2020_2990_MOESM1_ESM.docx]

**Supplementary materials**

**Supplementary Table 1 –** Proportion of potential drug-drug interactions associated with serotonergic syndrome involving co-administration of serotonergic medications and linezolid in FAERS database.

**Supplementary Table 2 –** Proportion of serotonin syndrome (SS) reports and mean number of drug-drug interactions (DDIs) reported with serotonergic agents when co-administered with linezolid in FAERS database. Red, yellow, and green zones identify respectively high-risk, intermediate-risk, and low-risk medications based on threshold values of ≥ 0.10% for proportion of SS reports and ≤ 1.5 for mean number of DDIs. For medications showing grey zone, risk was not assessed (number of reports < 5 and proportion of SS reports < 0.03%).

**Supplementary Table 3 –** Summary of pharmacokinetic and pharmacodynamic features of serotonergic agents.

**~~Supplementary Figure 1 –~~** ~~Scatterplot showing the relationship between V~~_~~D~~_~~/Ki SERT ratio (PK/PD index; x-axis) and mean number of DDIs (PV index; y-axis). A significant correlation was found (ρ= -0.53; p= 0.05).~~

**Supplementary Table 1 –** Proportion of potential drug-drug interactions associated with serotoninergic syndrome involving co-administration of serotonergic medications and linezolid in FAERS database.

| **Drug-drug interaction** | **Proportion of reports in serotonin syndrome** |
| --- | --- |
| ***Single DDI*** | |
| Linezolid-Citalopram | 69 |
| Linezolid-Fentanyl | 46 |
| Linezolid-Escitalopram | 45 |
| Linezolid-Sertraline | 40 |
| Linezolid-Venlafaxine | 21 |
| Linezolid-Tramadol | 20 |
| Linezolid-Methadone | 17 |
| Linezolid-Mirtazapine | 15 |
| Linezolid-Fluoxetine | 15 |
| Linezolid-Paroxetine | 13 |
| Linezolid-Metoclopramide | 13 |
| Linezolid-Amitriptyline | 11 |
| Linezolid-Duloxetine | 9 |
| Linezolid-Meperidine | 6 |
| Linezolid-Bupropion | 4 |
| Linezolid-Rasagiline | 4 |
| Linezolid-Oxitriptan | 3 |
| Linezolid-Trazodone | 3 |
| Linezolid-Buspirone | 3 |
| Linezolid-Hydromorphone | 2 |
| Linezolid-Morphine | 1 |
| Linezolid-Fluvoxamine | 1 |
| Linezolid-Nortriptyline | 1 |
| Linezolid-Desvenlafaxine | 1 |
| Linezolid-Modafinil | 1 |
| Linezolid-Entacapone | 1 |
| Linezolid-Dextromethorphan | 1 |
| ***Multiple DDIs (2 serotoninergic agents)***** | |
| Linezolid-Citalopram-Tramadol | 23 |
| Linezolid-Escitalopram-Trazodone | 11 |
| Linezolid-Fluoxetine-Metoclopramide | 10 |
| Linezolid-Methadone-Ritonavir* | 8 |
| Linezolid-Duloxetine-Fentanyl | 7 |
| Linezolid-Imipramine-Venlafaxine | 6 |
| Linezolid-Duloxetine-Trazodone | 5 |
| Linezolid-Amitriptyline-Fentanyl | 4 |
| Linezolid-Fentanyl-Metoclopramide | 4 |
| Linezolid-Meperidine-Buspirone | 4 |
| Linezolid-Duloxetine-Bupropion | 4 |
| Linezolid-Citalopram-Mirtazapine | 4 |
| Linezolid-Fentanyl-Fluoxetine | 3 |
| Linezolid-Paroxetine-Citalopram | 3 |
| Linezolid-Bupropion-Sertraline | 2 |
| Linezolid-Amitriptyline-Paroxetine | 2 |
| Linezolid-Metoclopramide-Tryptophan | 2 |
| Linezolid-Citalopram-Trazodone | 2 |
| Linezolid-Mirtazapine-Paroxetine | 2 |
| ***Multiple DDIs (3 serotoninergic agents)****** | |
| Linezolid-Bupropion-Trazodone-Sertraline | 7 |
| Linezolid-Trazodone-Fentanyl-Escitalopram | 6 |
| Linezolid-Fentanyl-Fluoxetine-Tramadol | 6 |
| Linezolid-Fentanyl-Trazodone-Sertraline | 3 |
| Linezolid-Citalopram-Trazodone-Hydromorphone | 2 |
| Linezolid-Fentanyl-Oxycodone-Citalopram | 2 |
| Linezolid-Desipramine-Sertraline-Imipramine | 2 |
| ***Multiple DDIs (4 or more serotoninergic agents)***** | |
| Linezolid-Fluoxetine-Sertraline-Fluvoxamine-Fentanyl | 13 |
| Linezolid-Fentanyl-Morphine-Venlafaxine-Oxycodone-Sertraline | 1 |
| Linezolid-Meperidine-Sertraline-Tramadol-Metoclopramide | 1 |
| Linezolid-Sertraline-Amphetamine-Fluvoxamine-Desvenlafaxine-Methamphetamine-Buprenorphine | 1 |
| Linezolid-Ondansetron-Citalopram-Trazodone-Hydromorphone | 1 |
| Linezolid-Metoclopramide-Meperidine-Droperidol-Fentanyl-Ondansetron | 1 |
| Linezolid-Fluoxetine-Propoxyphene-Trazodone-Metoclopramide-Meperidine | 1 |

* Linezolid-Ritonavir: pharmacokinetic interaction

** One case for: Linezolid-Escitalopram-Mirtazapine; Linezolid-Granisetron-Ondansetron; Linezolid-Fentanyl-Codeine; Linezolid-Meperidine-Metoclopramide; Linezolid-Escitalopram-Duloxetine; Linezolid-Tramadol-Propoxyphene; Linezolid-Methadone-Fluoxetine; Linezolid-Citalopram-Sumatriptan; Linezolid-Meperidine-Buspirone; Linezolid-Citalopram-Ritonavir; Linezolid-Ondansetron-Hydromorphone; Linezolid-Clomipramine-Trazodone; Linezolid-Fentanyl-Meperidine; Linezolid-Venlafaxine-Bupropion; Linezolid-Venlafaxine-Sertraline; Linezolid-Fluoxetine-Imipramine; Linezolid-Sertraline-Amitriptyline; Linezolid-Paroxetine-Amitriptyline

*** One case for: Linezolid-Citalopram-Venlafaxine-Fentanyl; Linezolid-Citalopram-Hydromorphone-Methadone; Linezolid-Trazodone-Metoclopramide-Mirtazapine; Linezolid-Sertraline-Tramadol-Meperidine; Linezolid-Bupropion-Trazodone-Venlafaxine

**Supplementary Table 2 –** Proportion of serotonin syndrome (SS) reports and mean number of drug-drug interactions (DDIs) reported with serotonergic agents when co-administered with linezolid in FAERS database. Red, yellow, and green zones identify respectively high-risk, intermediate-risk, and low-risk medications based on threshold values of ≥ 0.10% for proportion of SS reports and ≤ 1.5 for mean number of DDIs. For medications showing grey zone, risk was not assessed (number of reports < 5 and proportion of SS reports < 0.03%).

| **Serotoninergic agent** | **Absolute number of reports** | **Proportion of SS reports** | **Mean number of DDIs** | **Risk zone** |
| --- | --- | --- | --- | --- |
| **Citalopram** | 112 | 0.277% | 1.45 |  |
| **Escitalopram** | 65 | 0.250% | 1.48 |  |
| **Sertraline** | 74 | 0.095% | 2.12 |  |
| **Paroxetine** | 22 | 0.035% | 1.50 |  |
| **Fluoxetine** | 50 | 0.064% | 2.40 |  |
| **Fluvoxamine** | 15 | 0.249% | 3.93 |  |
| **Venlafaxine** | 33 | 0.056% | 1.58 |  |
| **Desvenlafaxine** | 2 | 0.012% | 3.50 |  |
| **Duloxetine** | 26 | 0.044% | 1.65 |  |
| **Bupropione** | 19 | 0.035% | 2.21 |  |
| **Mirtazapine** | 23 | 0.089% | 1.48 |  |
| **Trazodone** | 44 | 0.226% | 2.59 |  |
| **Buspirone** | 8 | 0.101% | 1.63 |  |
| **Desipramine** | 2 | 0.054% | 3.00 |  |
| **Amitriptyline** | 19 | 0.088% | 1.42 |  |
| **Imipramine** | 9 | 0.146% | 2.22 |  |
| **Clomipramine** | 1 | 0.014% | 2.00 |  |
| **Nortriptyline** | 1 | 0.015% | 1.00 |  |
| **Fentanyl** | 100 | 0.104% | 2.06 |  |
| **Oxycodone** | 4 | 0.004% | 3.50 |  |
| **Morphine** | 2 | 0.003% | 3.00 |  |
| **Tramadol** | 51 | 0.122% | 1.82 |  |
| **Buprenorphine** | 1 | 0.002% | 6.00 |  |
| **Propoxyphene** | 2 | 0.027% | 3.50 |  |
| **Methadone** | 27 | 0.108% | 1.41 |  |
| **Codeine** | 1 | 0.007% | 2.00 |  |
| **Meperidine** | 17 | 0.184% | 2.18 |  |
| **Hydromorphone** | 8 | 0.021% | 2.50 |  |
| **Metoclopramide** | 34 | 0.117% | 1.88 |  |
| **Droperidol** | 1 | 0.054% | 5.00 |  |
| **Dolasetron** | 1 | 0.276% | 4.00 |  |
| **Granisetron** | 1 | 0.036% | 2.00 |  |
| **Ondansetron** | 5 | 0.027% | 3.40 |  |
| **Sumatriptan** | 1 | 0.004% | 2.00 |  |
| **Oxitriptan** | 3 | 4.545% | 1.00 |  |
| **Rasagiline** | 4 | 0.143% | 1.00 |  |
| **Methamphetamine** | 1 | 0.025% | 6.00 |  |
| **Amphetamine** | 1 | 0.028% | 6.00 |  |
| **Tryptophan** | 2 | 0.980% | 2.00 |  |
| **Modafinil** | 1 | 0.020% | 1.00 |  |
| **Dextromethorphan** | 1 | 0.013% | 1.00 |  |
| **Ritonavir** | 9 | 0.041% | 2.00 |  |
| **Entacapone** | 1 | 0.016% | 1.00 |  |

SS: serotonin syndrome; DDIs: drug-drug interactions

**Supplementary Table 3 –** Summary of pharmacokinetic and pharmacodynamic features of serotonergic agents.

| **Concomitant drug** | **SERT IC_50_ (nM/L)** | **SERT Ki (nM/L)** | **5-HT_2A_ Ki (nM/L)** | **C_max_**  **(nM/L)** | **AUC**  **(mg/L*h)** | **V_D_**  **(L/kg)** | **LogP** | **Dose (mg/day)** |
| --- | --- | --- | --- | --- | --- | --- | --- | --- |
| **Citalopram** | 1,16 | 1,1 | 5600 | 385,34 | 685 | 33 | 3.76 | 40--60 |
| **Escitalopram** | 1,1 | 0,8 | 1000 | 198,5253023 | 1100,9 | 26 | 3.76 | 10--30 |
| **Paroxetine** | 0,29 | 0,065 | 6320 | 176,0962141 | 540 | 16 | 3.15 | 20--60 |
| **Fluvoxamine** | 3,8 | 11 | 12000 | 446,0639568 | 873 | 25 | 2.89 | 200-300 |
| **Fluoxetine** | 6,8 | 5,7 | 141 | 1070,068126 | 1997 | 12 | 4.09 | 20--80 |
| **Sertraline** | 0,19 | 2,8 | 2207 | 104,4966202 | 546 | 25 | 5.06 | 100 |
| **Venlafaxine** | 8,9 | 7,5 | 100000 | 316,6201851 | 5959 | 83 | 2.69 | 202 ± 79,3 |
| **Duloxetine** | 28 | 4,6 | 504 | 134,8284384 | 591 | 25,7 | 4.72 | 40--60 |
| **Bupropion** | 19000 | 9100 | 100000 | 700,7562328 | 1744 | 19 | 3.28 | 100--300 |
| **Mirtazapine** | 100000 | 100000 | 16,4 | 857,3488362 | 808 | 4,84 | 3.21 | 15--80 |
| **Trazodone** | 160 | 252 | 20 | 2877,396037 | 6320 | 1,08 | 2.68 | 50 |
| **Amitriptyline** | 4,3 | 2,8 | 5,3 | 527,3909863 | 661 | 20,4 | 5.1 | 75-200 |
| **Imipramine** | 35 | 8,7 | 119 | 1868,710265 | 427 | 16,9 | 4.28 | 200--250 |
| **Tramadol** | 3,3 | 1,19 | 13000 | 2,5 | 10473 | 4,37 | 2.71 | 100--400 |
| **Fentanyl** | 154 | - | 1300 | 0,03 | 820 | 8,4 | 4.12 | 0,012--0,2 |
| **Meperidine** | 1,6 | - | 3600 | 6 | 522,7 | 4,4 | 2.9 | 50--150 |
| **Methadone** | 0,23 | - | 610 | 7,3 | 6330 | 13,4 | 5.01 | 60--120 |
| **Hydromorphone** | 1000 | - | - | 69,04099949 | 123,1 | - | 1.69 | 48 ± 11 |
| **Metoclopramide** | 30000 | - | - | 6671,203085 | - | - | 2.18 | 4,5--14* |

* mg/kg/day; Ki: binding affinity; IC_50_: concentration corresponding to 50% inhibition of activity in vitro; C_max_: peak concentration; AUC: area under plasma concentration curve; V_D_: volume of distribution. For C_max_, AUC, and V_D_ the highest mean value reported for each medication when administered at therapeutic dosage was selected.
